# Who’s that girl? A qualitative analysis of adolescent girls’ views on factors associated with teenage pregnancies in Bolgatanga, Ghana

**DOI:** 10.1186/s12978-016-0161-9

**Published:** 2016-04-14

**Authors:** J. K. Krugu, F. E. F. Mevissen, A. Prinsen, R. A. C. Ruiter

**Affiliations:** Department of Work and Social Psychology, Maastricht University, Maastricht, Netherlands; Adolescent Sexual & Reproductive Health Unit, Youth Harvest Foundation, Bolgatanga, Ghana

**Keywords:** Adolescents, Teenage pregnancy, Contraception, Condom use, Determinants, Ghana

## Abstract

**Background:**

Adolescent pregnancy remains a public health concern, with diverse serious consequences, including increased health risk for mother and child, lost opportunities for personal development, social exclusion, and low socioeconomic attainments. Especially in Africa, teenage pregnancy rates are high. It is important to find out how girls without pregnancy experience differ in their contraceptive decision-making processes as compared with their previously studied peers with pregnancy experience to address the high rate of teenage pregnancies.

**Methods:**

We conducted semi-structured in-depth interviews with never been pregnant girls (*N* = 20) in Bolgatanga, Ghana, to explore the psychosocial and environmental factors influencing the sexual decision making of adolescents. Themes such as relationships, sex, pregnancy, family planning and psychosocial determinants (knowledge, attitudes, self-efficacy, norms, risk perceptions) derived from empirical studies and theories related to sexuality behavior guided the development of the interview protocol.

**Results:**

Results showed that the girls did talk about sexuality with their mothers at home and did receive some form of sexual and reproductive health education, including the use of condoms discussions in school. Participants reported high awareness of pregnancy risk related to unprotected sex, were positive about using condoms and indicated strong self-efficacy beliefs towards negotiating condom use. The girls also formulated clear future goals, including coping plans such as ways to prevent unwanted pregnancies to reach these targets. On the other hand, their attitudes towards family planning (i.e., contraceptives other than condoms) were negative, and they hold boys responsible for buying condoms.

**Conclusion:**

An open parental communication on sexuality issues at home, comprehensive sex education in school and attitude, self-efficacy, risk perception towards contraception, alongside with goal-setting, seem to be protective factors in adolescent girls’ pregnancy prevention efforts. These factors should be targets in future intervention programs at the individual, interpersonal, and school and community levels.

## Background

The present generation of adolescents is the largest in history. Nearly 90 % live in low-and-middle-income countries where they constitute a far greater proportion of the total population because of the higher fertility rates as compared to high-income countries [1, 2]. Most of these youngsters are sexually active [3, 4], thus putting a significant number of the world population at risk for getting infected with sexually transmitted diseases or getting pregnant unintentionally.

Adolescent pregnancy is a recognized public health concern that has diverse consequences for the individual, the family, and society as a wholee [5, 6]. Across sub-Saharan Africa, it is estimated that 14 million unintended pregnancies occur every year, with almost half occurring among women aged 15–24 years [7]. In Ghana, the Reproductive and Child Health Department of the Ghana Health Services reported a rise in adolescent pregnancies among those aged 15 to 19 years from 43,465 in 2009 to 83,917 in 2013, representing 12.3 % of all pregnancies in the country [8]. In rural areas like the Upper East Region, 21.7 % of adolescents aged 15 to 19 years had begun childbearing in 2014 as compared to 8.3 % in the Greater Accra region [9].

Teen pregnancy contributes significantly to the high maternal mortality rate of 920 deaths per 100,000 live births in Africa [6]. Among the 14.3 million adolescent girls that gave birth in 2008, one out of three was from sub-Saharan Africa and pregnancy-related morbidity and mortality rates are particularly high in this group [10]. The bodies of adolescent girls are not yet sufficiently developed to deal with pregnancy in a healthy way, and this age group is twice as likely to die in childbirth as women aged 20 or over [11]. Also, across Africa, one-quarter of the estimated 6 million unsafe abortions and 22,000 abortion-related deaths each year occur among women aged 15–19 years, showing that most of the pregnancies among teenagers are unwanted [6].

While the use of effective contraception can prevent unwanted or unplanned pregnancies, few sexually active adolescents use contraceptives methods such as hormonal contraceptives (often referred to as ‘family planning’) and condoms. For example, among sexually active Ghanaian adolescents age 15 to 19 years, 80 % of girls and 63 % of boys were not using any contraceptive method at their last sexual encounters [12]. An earlier report also showed that only 10 –12 % of adolescents’ aged 15 –19 years who were not using contraceptives intended to do so in the next 12 months [13].

Addressing the high rate of teenage pregnancies and non-contraceptive use in Africa requires a context-specific understanding of why teen pregnancy is so high among adolescents while a significant group does seem to manage to prevent getting pregnant. Previously identified barriers to contraceptive use among adolescents in sub-Saharan Africa include inadequate sexual knowledge and risk perceptions, lack of skills to negotiate safer sex options, ambivalent attitudes towards sex, lack of access to educational and health services, and negative social norms around premarital sexual activity and pregnancy [Krugu, Mevissen, Meret & Ruiter, under review, [14, 15]]. Other empirical reports also indicate that young girls may engage in unsafe sex because they have not considered contraception [[16], Krugu et al., under review], are afraid of possible side effects [17], are more worried about the safety of contraceptives than preventing an unintended pregnancy [18], or are not adequately informed about the risk of pregnancy or disease posed by unsafe sex [19].

However, most of these studies have focused on pregnant girls and those with pregnancy experience – following the general long held practice of merely focusing on people with the health problems. There is limited work on never-pregnant adolescent girls’ perceptions regarding pregnancy prevention. Gaining an understanding of adolescent pregnancy from the perspective of never-pregnant teenage girls can play a major role in identifying factors that influence sexual decision-making in young women regarding initiating sexual activity and preventing pregnancy. Studying girls without pregnancy experience could also provide pointers on the capacity and needs that girls with pregnancy experience are lacking, which, in turn, facilitates the development of intervention goals. Knowledge of the perceptions, beliefs and behaviors of never-been-pregnant adolescent girls is, therefore, necessary to guide the content of future teenage pregnancy prevention programs.

To fill this gap, we conducted the present study as part of a larger research project exploring the behavioral, environmental and psychosocial factors influencing adolescent sexual and reproductive health behaviors. More specifically, the project was initiated to create a comprehensive picture of factors associated with the high teenage pregnancy rates in Northern Ghana and to inform future prevention strategies. After a study focusing on teenage girls *with* pregnancy experience in Bolgatanga, Ghana (Krugu et al., under review), the current study reports on the qualitative interviews conducted among adolescent girls *without* pregnancy experience. The aim was to explore the psychosocial and environmental factors influencing their sexual decision-making, which seems to put them not or less at risk of unintended pregnancies as compared to their peers with pregnancy experience.

## Methods

### Study design

The study used semi-structured individual in-depth interviews to explore the behavioral, environmental and psychosocial determinants of sexual choices of adolescent girls who do not have pregnancy experience. The semi-structuring helped to ensure consistency across interviewers and interviewees. The Ethics Committee of the Ghana Health Services in Accra and the Ethics Review Board of Maastricht University in the Netherlands approved the study.

### Study setting

We conducted the study in the Bolgatanga Municipality in northern Ghana. Bolgatanga is located 743.83 km to the north of Accra (capital of Ghana) and covering a total land area of 729 sq km^2^. Females make up 52.3 % of an estimated population of 131,550 and 44 % are below 18 years with a growth rate of 3.0 % [20]. Agriculture is the main economic activity in the Bolgatanga Municipality, and it is among the poorest districts in the country with 35 % of its population living on less than the World Bank’s threshold of $1.25 per day [21]. The healthcare system in the Municipality includes a Regional Hospital, nine Health Centres, and several Community-based Health Planning and Services (CHPS) compounds.

### Study population

Our final sample included 20 adolescent girls between the ages of 14 and 19 years (M = 17.3, SD = 1.59) with no pregnancy experience and living in the Bolgatanga Municipality of the Upper East region of Ghana. Six participants reported sex experience; 3 of them were 18 years of age and the other 3, 19 years. The girls came from families engaged in peasant farming or doing petty trading businesses. The girls lived together with siblings in larger families – an average of 5 children per family. Two girls had lost their fathers, and one had lost her mother. One girl came from a polygamous family, and another one had a Burkinabe father and a Ghanaian mother. All the participants were still in various stages of high school. The sample included 5 Muslims and 15 Christians, and all of them indicated that religion was important to them. Although all the girls were from the low socio-economic background, they all had clear goals and hopes of a better future career and indicated their preparedness to work towards such goals.

### Recruitment and Procedures

The study used a purposeful homogeneous sampling technique to recruit the participants [22]. A short description of the study, including the purpose, the voluntary nature of participation and how to register to participate, was advertised in schools and other public places in Bolgatanga where young girls are likely to visit. Also, research assistants visited schools to recruit girls. Interested participants confirmed their participation by completing an informed consent form and those below the age of 18 years took the form home for parental consent before they could participate. Participants were enrolled day-by-day until thematic saturation was reached.

In all, 28 girls signed up for the study, of which 25 met the criteria of being adolescent (10 ≤ years old ≤ 19) and having no pregnancy experience. The 25 girls were then asked to complete informed consent forms and for those below age 18 years (*N* = 8), research assistants visited their homes to explain the study and secured additional parental consent. In the end, we interviewed 23 girls who returned completed forms to participate. We did not compensate for participation, but where necessary, participants’ transport costs were paid, and all received a soda drink at the beginning of each interview. The interviews lasted between 45 minutes and 1 hour. All interviews were audiotape recorded, and after transcription, the tapes were erased.

Researching sensitive issues such as sexuality in a culturally inhibiting environment like northern Ghana necessitated an ethical awareness in the development of the research method. Anonymity and voluntary participation was warranted by not documenting identifiable details of participants and not applying any persuasion regarding participation. Also, the research team, was conscious of concerns that young girls in northern Ghana are in a position of both economic and social vulnerability [23]. To ensure that hierarchical research methods did not exploit the girls, the research assistants were trained to pose questions as supportive as possible and the interviews terminated if signs of distress were observed. At the beginning of each interview, participants were also assured of the confidential handling of the data to avoid that they provide socially acceptable responses. Finally, participants were informed of the option to quit the interview at any time necessary without having to provide an explanation.

Since the participants were all high school students who could speak and understand English, the interviews were mostly carried out in English; both the questions posed by the interviewer as well as the responses given by the participants. However, sometimes the research assistant (who spoke English but also was a native speaker of ‘Frafra’) had to translate questions posed in English by the lead interviewer for participants who could not understand some of the English formulations or questions. Also, sometimes participant’s responses were in Frafra if they had difficulties finding the correct English expression. These responses were also translated in the same manner to the lead interviewer, and we recorded both languages for the transcription. The first author, who speaks the local language as well, validated the transcriptions by listening to the voice recordings and made minor corrections where necessary. The participants determined the locations of the interviews. Out of the 23 individual interviews conducted, 3 were excluded from the analysis because of bad recording quality and/or interview stopping half-way because participants had to attend to parental calls.

### Research instrument

We used a semi-structured interview guide that was developed based on theoretical concepts and literature review. The interview protocol included themes related to knowledge, attitude, self-efficacy, risk perception, and social norms addressing topics such as relationships, sex experience, pregnancy, contraception, and sexuality communication. Table 1 presents the main themes and topics of the interview instrument. Two young trained women, a Ghanaian with prior training in youth peer sex education and a Dutch graduate student from Maastricht University, conducted and transcribed the interviews. The corresponding author validated the transcripts by listening to the interviews and making minor corrections where necessary.

**Table 1:** Interview protocol showing the themes and topics that guided the data collection

Theme/topics	Examples of subtopics	Examples of questions
Introduction	Age, background, school/work, family, life, religion, ethnic group.	Can you tell me something about yourself? What does your life look like? Do you go to school, work? What do you like to do in your free time? Can you tell me a bit about your family? Would you consider yourself being religious? What role does religion play in your life?
Background variables		
Demographics		
Pregnancy/motherhood	Pregnancy	How do you think about pregnancy? What do you think it would be like to be a mother? What would be a good time to become pregnant/a mother according to you? How would you feel if you found out you were pregnant now/at this moment in your life? What would you do if you figured out you were pregnant now. Do you know anyone of your age who got pregnant? What do you think of that? How do important others in your surrounding think about teenage pregnancy in general of ‘if it was you being pregnant’ How do you feel about their opinion?
	Motherhood	
	Important others include: mother, father, brother/sister, friends, boys/girls in your village or at your school, the teachers, and church leaders? Health centre workers?	
Femininity/masculinity		What does it mean to be a woman? What do you think are the main differences between men and women of your age? How do important others think about women?
Relationships	Current relationships	Are you in a relationship at the moment? How did your relationship start? How would you define your current relationship? Where do you meet your partner?
	Past relationships	
	First relationship	
		What do important others think about your relationship?
Sex experience	Attitude towards sex	Did you ever have sex? Why/why not? How many partners?
	Positive/negative experiences	If you have sex, where (At home? Church? Bushes? Parties? School?)
		How would you describe your sex life/how are your sex experiences? (positive/negative experiences)
		Do other people know you have sex? (why/why not). What do you think important others think about you having sex?
Safe sex	Definition and importance of sex	What do you consider safe sex?
	Definition of safe sex	Do you use condoms? (how often?)
	Contraceptive use	Do you use other types of contraception? Which one? What are reasons for using/not using condoms (or other forms of contraception)? Who do you think is responsible for contraception? What are advantages and disadvantages of condoms/contraception? What do you prefer (condom or other contraceptives) and why? Should men/women carry them around?
	Condom use	
	Where/how do they get condoms/other contraception	
Safe sex negotiation with partner		People don’t always want the same with sex. For example some want to use condom others don’t. Or some like sex in one way and others like it in different way. What do you do/would you do if partner want something else? What if you want to use a condom but he doesn’t?
		Have you ever persuaded a man to practice safe sex? Can you describe how you discussed it with him? What was the reason for not discussing?
Communication about (safe) sex with partner	Topics of communication with partner	Which topics can/can’t you discuss with partner?
	Taboo topics	Do you ever talk about sex with your friends/sisters/parents?
	Communication	
Sex education/sex communication	Information sources	Did you learn about sex? How/by whom? Did you have sex education at school? What did you think of it? Do you ever talk about sex, contraceptives, motherhood at school, with your friends, family? Do you think you know all you need to know about sex/safe sex? If you have questions (e.g. on condom/contraception/pregnancy) where would you go? Did you have to go to health clinic for sex-related issues? What is your experience?

### Data analysis

After validity checks and proof-reading, the 20 transcripts in MS Word documents were exported into NVivo 10.0 qualitative software for analysis. The analysis took the form of thematic exposition -- identifying the dilemmas, fears, and beliefs within the narratives [24]. A thematic exposition allows the researcher to determine categories and construct concepts using a grounded theory approach [25]. The analysis employed a three-level coding system. At the first level, the transcripts were repeatedly read to identify phrases which could be coded into general themes. We grouped these initial codes into smaller themes at the second level coding (axial coding or pattern coding). At the third or selective coding level, we reviewed level one and two codes to confirm the various thematic categories. The second author checked the three levels of codes [26] against the original transcripts and discussed a few little parts that were not covered under the right topics back-and-forth with the first author to reach an agreement. The thematic categories were then summarized and used as the basis of subordinate and secondary analysis of the determinants of adolescent girls’ sexual health decision-making processes in the Bolgatanga area as viewed from the perspective of unmarried teenagers without pregnancy experienced.

## Results

Table 2 presents the main themes that emerged from the data, and the number of girls who reported on the various thematic areas. We have classified the results according to behavioral factors, environmental factors, and psychosocial determinants related to sex and sexuality, contraceptive use, and teenage pregnancy. It seems there were some overlapping patterns in the girls’ responses. For example, those who reported a positive relationship with their parents also did express a desire to stay in school and had clear intentions to make safer sex choices to safeguard future plans.

**Table 2:** A matrix of the main themes that emerged from the data and the number of participants who reported those themes

Main theme	Number of girls reporting
Buying condoms	10
Carrying condoms and condom use responsibility	16
Attitude towards condoms and condom Use	17
Knowledge on condom use	20
Risk perception towards both pregnancy & STIs	19
Intention to use and negotiating condom use	15
Life situation of the girls (occupation, economic situation)	18
Knowledge on family planning/contraception	17
Attitude towards school/education	19
Attitude towards sex	9
First time sex experience	6
Experiences with forced sex	4
Social norms towards pregnancy	10
Sex education at the health facility	8
Sex education in school	17
Sex education/communication at home	14
Social norm towards adolescent sexuality	13
General relationship experiences	9
General sex experiences	6
Condom use experience	6
Future goals & its influence on safer sex choices	20
Intention to use condoms	9

### Behavioural factors

#### Relationship experience

Half of the girls mentioned that they had experienced relationships with boys and had pleasant and steady relationships lasting between one to six years. For most of them, their relationships were not secret but known to others, including their parents and they perceived that others were positive about the relationship. The girls indicated that not keeping the relationship secret was important to them because the boys cannot deny responsibility if they mistakenly get pregnant. One girl put it this way:

*P: We always do it plain, so people know. In that way, there can be no denying if anything happen*

*{18 yrs Muslim in Senior High School}*

Some of the girls are in a relationship because of potential help (material gain) from the boys as in:

*P: Like anytime I want money to do [anything] at school, he will give me the money, and I like him*

*{19 yrs old Christian in Senior High School}*

#### Sex experience

The six girls with sex experience were mostly one year older than those without sex experience, and most of them had been in the relationship for an average of one year before initiating sex. Concerning sexual debut, they reported mixed experiences. Some girls described their sexual debut as “good” or “somehow good” and for this category they had planned for it and also used condoms:

*I: When did you have sex for the first time? P: The first time it was a year of the day we met, that was our one year celebration. I: Did you talk about it with your boyfriend before? P: Yes I: Was it a good experience? P: Somehow good.*

*{19 yrs Christian in Senior High School}*

Others had bad experiences such as forced sex and painful intercourse:

*I: Can you tell me a bit about your first sexual experience? P: Oh it was very painful, very painful. I: how old were you when you had your first sexual experience? P: 16 years.*

*{18 yrs Christian in Senior High School}*

The girls who were sexually active said they initiated sex because of the perception that their peers were doing it. Some girls also indicated that they had encountered forced sex, and some had sex with their boyfriends’ because the boys’ had threatened to end the relationship if they did not yield to their request for sex. Some few girls also added that they had been approached by teachers demanding to have sex with them and they refused. They believed that most teachers are making similar advances towards young girls.

#### Condom use experience

All sexually experienced girls indicated using condoms consistently. Also, they all stated that they discussed sex and condom use with partners before sex. The girls were clear of the need to use condoms and they negotiated for it:

*P: Yes, I am using condoms with my partner because he has not married me yet and so far as he loves me, we can have sex. But if we do not use a condom, it is too bad. We have to use condoms to avoid unwanted pregnancy.*

*{19 yrs, Christian in Senior High School}*

### Environmental factors

#### (Sex) communication with parents and peers

The girls’ reported an attuned and warmth relationship with their parents (mostly mothers) at home. The cordial home environment seemed to have resulted in a relatively open mother-daughter communication. They also discussed exuality-related topics with their mothers. However, such discussions centered on moral advice and abstinence-only messages, except for a few girls who reported receiving safe sex practices advise. Many of them indicated that their parents discouraged them from thinking and talking about sexuality issues. When asked why they think their parents showed little interest in talking about sex with them, the majority indicated that their parents harbored fears that talking about sex will encourage them to practice more sex. One girl put it as follows:

*P: It is up to them, I don’t know. They think we shouldn’t be in love.*

*(19 yrs Christian in Senior High School)*

The girls reported that their mothers continuously admonished them to delay entering into sexual relationships or use protection against pregnancy. In general, the girls were not happy with the emphasis on abstinence-only messages from their mothers, but those who had more extensive advice on relationship and sex were glad to have such talks:

*P: she said that when you are having sex, you have to use condoms. I feel great that someone is concerned about my life.*

*{19 yrs Catholic in Senior High School}*

At the same time, some girls expressed their desire to discuss sexuality issues with their parents, but they missed such discussions:

*P: It is good they talk but when they don’t talk, what can you do? You cannot go and tell them, come here and sit and talk with me!*

*{17 yrs Christian in Senior High School}*

Some girls preferred to discuss matters of sexuality with peers instead of talking with their parents who will only preach morality to them. Among peers, the discussion often centered on advising each other about the “good and bad” side of their sexual lives:

*P: We talk about the bad effects of sex; we speak about the good part and the bad part. I: Hm, what are the good parts and what are the bad? P: The good part is when you marry and give birth to children then you are fulfilling God’s promise like that, but the bad effects is that you maybe a teenager too, you have it, and you get pregnant, who can take care of the child, that Is on the wrong side.*

*{18 yrs Christian in Junior High School}*

#### Experience with and Attitude towards sex education

The majority of the girls received sex education in school, which seemed to be skills-based and included condom use demonstration. They shared positive experiences with the school-based sex education:

*P: It was good, they told us about AIDS and teenage pregnancy and gonorrhea, syphilis, all this kind of disease, […] so they taught us all the things in school, including how to use condoms, yes.*

*{19 yrs Catholic in Senior High School}*

And:

*It was good […] if you are even at my age, and you want to have sex and they even teach you how to prevent yourself from those sickness, you now know how to keep yourself and how to have safe sex with a man.*

*{15 yrs Muslim girl in Junior High School}*

On the other hand, some of them got abstinence-only messages and one never got sex education at all. For some, nurses from the health education unit came to teach them in school and for others; it was either their teachers or peer educators from NGOs. On seeking information via health services, the majority of the girls had either already gone to the health facility for sex education or indicated that if they needed information on sexuality, they would go to the health center.

#### Psychosocial determinants

##### Determinants related to condom use

Except one girl who indicated her lack of knowledge, the girls reported that they were relative ly knowledgeable about condoms. In general, they knew about condoms and its use as a protection against unwanted pregnancies and STIs:

*P: Yeah, I told him that as young as we are, we have to use protectives like condoms, to avoid ourselves from being pregnant and he said okay. He also decided that we should use condoms, so that is why we use condoms*

*{19 yrs Catholic in Senior High School}*

Almost all the girls also seemed to know how to use condoms and, also, some knew that family planning (other contraceptives besides condoms) can also be used to prevent pregnancy. Most girls indicated that it was important for both boys and girls to carry condoms with them to be prepared for any unplanned sex:

*I: And what do you think if a girl carries condoms with her? P: I will think she wants to protect herself because you don’t know. Anything can happen at any time, so in case she is forced to have sex and want to protect herself, she can use it*

*{17 yrs, Christian in Junior High School}*

However, none reported ever buying condoms, including the girls with sex experience. While some of them do know that they can buy condoms at the drugstores, others seemed not to know exactly where to obtain condoms. The main reasons for not attempting to buy condoms included the belief that it is the boy’s responsibility to buy condoms or the perception that the store attendants will ridicule them:

*P: Because she’ll say who is this small girl coming to buy condoms, what are you going to use it for? But she’ll not ask you whether it’s for you or it’s not for you, they will just start saying that look at this small girl, and you are buying this thing to do what*

*{14 yrs Muslim in Junior High School}*

Regarding perceived behavioral control concerning negotiating condom use, the girls believed they were able to negotiate condom use with their partners by pointing out unwanted pregnancy and STIs as reasons why they must use condoms. They were prepared to stop the relationships if their partners do not agree to use protection. The following quote sums up the girls’ readiness to negotiate for condom use:

*P: When it happened that way I told him no condoms, no sex and he had to go out and bring one*

*{17 yrs old Christian at Senior High School}*

A common phrase “no condom, no sex” depicted how they were not willing to compromise on negotiating to use condoms.

The majority of the girls were positive about condoms and also about using condoms. They were clear about the importance of using condoms to protect against both pregnancy and STIs:

*P: Because if you use them well, you will not get pregnant and you will not get HIV/AIDS that is why I think condoms are good*

*{18 yrs, Christian girl in Junior High School}.*

Some gave reasons why other people may not be comfortable using condoms, such as a reduced sexual pleasure. The majority of the girls, including those without sex experience, were clear in their intentions to use condoms, and were prepared to negotiate for it or stop the relationship if the partner refuses (or would refuse) to use condoms:

*P: Okay, if it happens.... I don’t think I’ll agree to have sex without condoms, and if he doesn’t agree too, I’ll walk away from him*

*{17 yrs old Christian girl in Senior High School}*

The reasons for clear intentions to use condoms included being able to continue their education to achieve their goals, not wanting to become a disgrace to their family, and not wanting to live in poverty. In general, the girls were conscious of the risk that any girl can get pregnant at any time. The following quotes indicate their high level of perception of risk:

*P: me like this, I’m having my boyfriend. At this my age, maybe it can come to a mistake, then we sleep together, then I can get that pregnancy. But if you protect yourself, you’ll not get pregnant, but if you don’t protect yourself, if the boy release on you, you will get pregnant.*

*{18 yrs Catholic girl in Junior High school}*

A common phrase *“it can happen to any girl”* defined their perception of risk of pregnancy.

All the girls had clear goals towards the future, and the majority indicated they were concentrating on taking measures to ensure unintended pregnancy do not keep them from achieving their life goals. They wanted to become teachers, nurses, lawyers, doctors or business women. They reported measures such as using condoms and avoiding male friends to stay focused on achieving their goals.

*P: If you don’t stay away from boys now, but at my age like this, as of now I don’t have anything, I don’t have any work, then if I follow the boys in the end I’ll not get anything in the future, and my hope of becoming a nurse will fail. Maybe they can impregnate me now, and I’ll drop out from school.*

*{18 yrs, Christian in Junior High School}*

All the girls were very firm in their statements that they will only allow themselves to become pregnant and have children when they achieved their goals around the age of 25 to 30 years.

##### Determinants related to family planning

The girls defined family planning as contraceptives other than condoms. Thus, condom use was viewed separately from other family planning methods. All the girls were aware that condoms and family-planning can protect against unwanted pregnancy and, in the case of condoms, STIs as well. However, none of the girls was using a family planning method, and almost all of them believed that family planning is not right for unmarried girls. They gave reasons ranging from high potential of becoming infertile, growing slim or too fat, and falling sick to destroying your womb as in:

*P: I hear people talking about it that if you are doing it, it can destroy your womb, so you can’t give birth again, but they even said that it is only meant for those who are married.*

*{18 yrs Catholic girl at Junior High school}*

##### Determinants related to sex and pregnancy

The attitude of the girls towards sex is varied. To some, sex is a good thing

*I: When did you have sex the first time? P: Last year I: Okay, how was the experience? P: It was good experience, sex is good*

*{19 yrs Christian girl in Senior High School}*

To others, sex is only for married people based on what they heard from their parents or the church:

*P: Okay, as at my age, sex is not a good thing to me, but if you are a married woman, and honest, you have to have sex before you can give birth.*

*{18 yrs Catholic girl at Junior High school}*

Some of them said although sex is for married people, when they are not able to resist the urge and should have sex, they will protect themselves against pregnancy. Regarding social norms towards teenage pregnancy, the majority of the girls think that the important referent people in their lives consider teenage pregnancy as a bad thing to happen:

*P: They will say oh, look at this girl, she is pregnant, they will even be talking, everywhere about you, you are having pregnancy and what and what, your friends will now be afraid of you and not follow you again, now that you are a bad girl, that is why it is not good.*

*{16 yrs Muslim girl in Junior High school}*

On social norms towards girls being in sexual relationships, some of the girls indicated that other important referents to their lives, including their parents, were open to adolescent sexuality and relationships. They clearly indicated that their parents were aware that they are in sexual relationships, and some got advice on protection against pregnancy from their mothers. Other girls were of the view that their parents and other relative do not want to hear that they are in a relationship or have sexual relations with boys:

*P: It’s up to them, I don’t know. They think we shouldn’t be in love*

*{19 yrs Catholic girl in Senior High School}*

## Discussion

Knowledge of what makes girls without pregnancy experience differ from girls with (unintended) pregnancy experience can greatly enhance the development of effective pregnancy prevention programs. In this study, we interviewed adolescent girls living in communities with high teenage pregnancy rates but without pregnancy experiences themselves. The results suggest that the girls had a cordial and positive relationship with their parents and did talk about sexuality with their mothers and friends. Although much of the mother-daughter communication was largely limited to moral advice to abstain from sexual relationships, some girls received more extensive advice, including safer sex practices to avoid unwanted pregnancy. In school, the majority of the girls seemed to have received sex education with the inclusion of condom use. The girls also reported high awareness of the risk of pregnancy through unprotected sexual intercourse. They were also positive about carrying condoms (some girls) and using condoms. All the girls also indicated strong self-efficacy beliefs towards negotiating condom use. On the other hand, the girls believed that it is the boys’ responsibility to buy condoms, and they had negative attitudes towards so-called family planning (contraceptives other than condoms). There were some overlaps in the narratives of the girls. However, conclusions cannot be made base on these overlapping reporting of only 20 qualitative interviews. Whether there are potential relationships between the reports of the girls among the emerged themes will require a quantitative survey involving a statistically significant sample.

The results also suggest that the girls had clear future goals regarding educational levels and careers they want to achieve, combined with clear plans on how to achieve them. The latter mostly focused on preventing any pregnancy before having finished education and before having a proper job by staying away from boys or refusing any sex without condoms. All the girls had clear intentions for future condom use and those who were sexually active reported consistently using condoms to avoid pregnancy until the achievement of their plans.

Our participants’ experiences of frequently receiving advice on sex and relationship issues from their mothers are contrary to what was reported by Krugu et al. (under review), where the pregnancy experienced girls had no form of sex communication at home. Parents who keep the communication lines open all the time have been reported to have a closer and more connected relationship with their children, which allows them also to discuss sexuality topics [27, 28]. Although some of our participants’ experiences were largely that of being dissuaded from having sex, those who received more extensive advice, including condom use discussions, reported being happy to know how to “have sex with a man”. Which might explain why they could avoid unwanted pregnancy as compared to the pregnancy experienced girls of Krugu et al. (under review). Past research suggests that mother–adolescent discussion about condoms before adolescent’s sexual initiation was associated with more use of condoms at sexual initiation and could set the stage for later consistent condom use [29]. However, factors such as lack of time, lack of knowledge, not being comfortable and perceiving that their daughters are not at risk of pregnancy or STIs have been reported to inhibit mother-daughter communication on safe sex practices [28, 30]. Further exploration into the influences of mother-daughter sex communication is necessary to support family based interventions to address teenage pregnancy in North Ghana.

In contrast, to the girls with pregnancy experience (Krugu et al., under review) the girls in the current study all reported having had some form of school-based sex education that included lessons on the use of condoms. Across Africa, formal school-based sex education programs focus on promoting abstinence-only messages [31]. However, comprehensive programs also provide information on birth control methods and condoms to prevent both pregnancy and STIs [32]. These different topics included in sex education across schools could explain the differences in safer sex choices made by the girls without pregnancy experience as compared to the girls with pregnancy experience. Systematic reviews suggest that the effects of abstinence-only programs in reducing sexual risk behaviors have been minimal [33–35]. Rather, adolescents who received comprehensive sex education had a lower risk of pregnancy than those who received abstinence-only or no sex education [36].

The girls in this study reported positive attitudes towards condom use, exhibited high condom use self-efficacy, high-risk perceptions towards pregnancy, and had clear intentions towards condom use as compared to the girls who became pregnant against their wish (Krugu et al., under review). Some studies across Africa have demonstrated the role of attitude, self-efficacy beliefs, risk perception and subjective norm in shaping young people’s intention to use condoms [37–39]. Future research that seeks to confirm the relevance of these personal determinants in condom use decision-making among the adolescent population in northern Ghana is necessary to guide intervention development.

The girls in this study mentioned specific plans such as using condoms to prevent unwanted pregnancies that may threaten future goals. This is in contrast to Krugu et al.’s (under review) study of girls with pregnancy experience. The girls with pregnancy experience often described and perceived themselves as being subordinate to boys. They also seemed not to have specific future goals. The current finding adds a potential new dimension to the efforts to prevent teenage pregnancy in Ghana. Several studies have shown the positive effects on health behavior and decision making of having clear future goals or purpose for living [40–43]. Our finding suggests that the proactive pregnancy preventive behaviors of adolescent girls to fulfill their aims could influence whether or not they will become victims of unintended pregnancy. Thus, it could be useful to get young girls to relate safe sex with life goals that they can forfeit by the outcomes of unsafe sexual activities [44]. At the same time, however, within the African context, goal-setting as a strategy to increase health-related decision making of girls may be difficult to accomplish. Across rural Africa, the choices of young women are often constrained by their narrow range of experiences, and they frequently project for themselves a life similar to that of their mothers, which is often characterized by low income, and limited economic opportunities [45].

A concerning finding is that, similar to the girls with pregnancy experience (Krugu et al., under review), the girls in the current study also had strong negative attitudes towards family planning (i.e. using other methods of contraception besides condoms) and believed that boys are responsible for buying and carrying condoms. These beliefs mostly put boys in control of safer sex choices. Therefore, although the girls in this study reported clear intentions to use condoms, they may, in the end, fail to act by their intentions. Programs aimed at promoting condom use need to stress that the importance of being prepared and in control or having condoms available is also important for girls. In addition, the ‘double Dutch’ approach (using another contraceptive method in addition to condoms) has been shown to be safer for pregnancy prevention than only relying on condom use [46]. Future research should explore strategies to change girls’ negative beliefs about using family planning outside marriage context.

### Study Limitations

Since all the girls belong to the ‘frafra’ ethnic group living in Bolgatanga, the findings are not necessarily generalizable to other African populations. However, the findings offer interesting perspectives to the discourse on adolescent girls’ sexual behaviors in Ghana and should be further confirmed through quantitative methods. Also, fearing that age difference between interviewers and participants may inhibit open discussion on sexuality, we used young women to collect the data. The interviewers had limited experience in conducting in-depth interviews on sensitive topics like sexuality. They might have had difficulty in determining whether or not a participant was responding in a socially desirable manner. More experienced interviewers could have enriched the data through the right follow-up questions or further probing. Also, the context in which we conducted the interviews could have influenced the results. A few times, interview sessions had to be moved to a more quiet location in the course of interviewing and the disruption led to some questions remaining unanswered. Our recruitment procedure required participants less than 18 years of age to obtain parental consent. It is possible that only parents who accepted that their daughter’s can discuss sexual topics openly consented, thus causing a bias in the generalizability of our sample. The recruitment procedure also ended up with only school-going girls as participants as well as with a sample of which only a limited number had sexual experiences. Both could have influenced our findings. Since the school setting can define the sexual socialization of girls in different ways [47], it is possible that the results would have differed if out of school girls were included in the sample. For girls without sexual experience, the answers on how they would deal with actual sex-related situations are therefore hypothetical.

## Conclusion

This study suggests that a more positive mother-daughter communication at home may be one of the protective factors in the sexual decision-making processes of girls. Since previous research also showed that mothers seem to be important socializing agents in adolescent sexual decision-making [27, 48], interventions may focus on helping the maternal parent become skilled, comfortable and open in discussions about sexuality with their daughters. Also, it seemed that access to school-based sex education that includes condom use information made a difference between the girls with pregnancy experience and those reported in this study. Therefore, the parental contribution could also include engaging in school policy development to ensure that school-based sexuality education goes beyond abstinence-only messages to equip adolescents for safer sexual choices.

Our results also point out how the attitude towards contraception, risk perception towards pregnancy, and girls condom use self-efficacy can influence the performance of action specific sub-behaviors necessary for girls’ safer sex practices. Preparatory sub-behaviors such as buying and carrying contraceptives and skills to communicate contraceptive use wishes are vital to enable girls to make safer sex choices [49]. Future intervention planners should first examine which cognitive beliefs are more important in addressing specific preparatory behaviors related to condom use. Such efforts may consider the ‘double-dutch’ approach of promoting the combined use of both condoms and hormonal contraceptives to enable girls to take full control of their sexuality [46].

Finally, this study suggests that girls with higher purpose may tend to use more pregnancy preventive measures to avoid unintended pregnancy and stay focused on achieving their life goals. Intervention studies designed to improve goal-setting and experiences of purpose in life may be warranted. Doing so could offer new avenues for girls’ increased use of pregnancy preventive measures, thereby decreasing the chances that they will become pregnant against their wish.
